# Solution-mediated nanometric growth of α-Fe_2_O_3_ with electrocatalytic activity for water oxidation†

**DOI:** 10.1039/d0na00345j

**Published:** 2020-07-20

**Authors:** Asako Taniguchi, Yuta Kubota, Nobuhiro Matsushita, Kento Ishii, Tetsuo Uchikoshi

**Affiliations:** Graduate School of Pure and Applied Sciences, University of Tsukuba 1-1-1 Tennodai Tsukuba Ibaraki 305-8573 Japan s1930108@s.tsukuba.ac.jp; Research Center for Functional Materials, National Institute for Materials Science (NIMS) 1-2-1 Sengen Tsukuba Ibaraki 305-0047 Japan; Department of Materials Science and Engineering, School of Materials and Chemical Technology, Tokyo Institute of Technology 2-12-1 Ookayama, Meguro-ku Tokyo 152-8550 Japan

## Abstract

This paper describes a simple, low-temperature, and environmentally friendly aqueous route for the layer-by-layer nanometric growth of crystalline α-Fe_2_O_3_. The formation mechanism involves alternative sequences of the electrostatic adsorption of Fe^2+^ ions on the surface and the subsequent onsite oxidation to Fe^3+^. A combination analysis of X-ray diffraction, scanning electron microscopy, UV-Vis spectroscopy, and X-ray photoelectron spectroscopy revealed that α-Fe_2_O_3_ is directly formed without post-growth annealing *via* designed chemical reactions with a growth rate of *ca.* 1.7 nm per deposition cycle. The obtained α-Fe_2_O_3_ layer exhibits electrocatalytic activity for water oxidation and, at the same time, insignificant photo-electrocatalytic response, indicating its defective nature. The electrocatalytic activity was tailored by annealing up to 500 °C in air, where thermal diffusion of Sn^4+^ into the α-Fe_2_O_3_ lattice from the substrate probably provides an increased electrical conductivity. The subsequent surface-modification with Ni(OH)_2_ lowers the overpotential (250 mV at 0.5 mA cm^−2^) in a 1 M KOH solution. These findings open direct growth pathways to functional metal oxide nanolayers *via* liquid phase atomic layer deposition.

## Abstract 2

This paper describes a simple, low-temperature, and environmentally friendly aqueous route for the layer-by-layer nanometric growth of crystalline α-Fe_2_O_3_.

## Graphical abstract

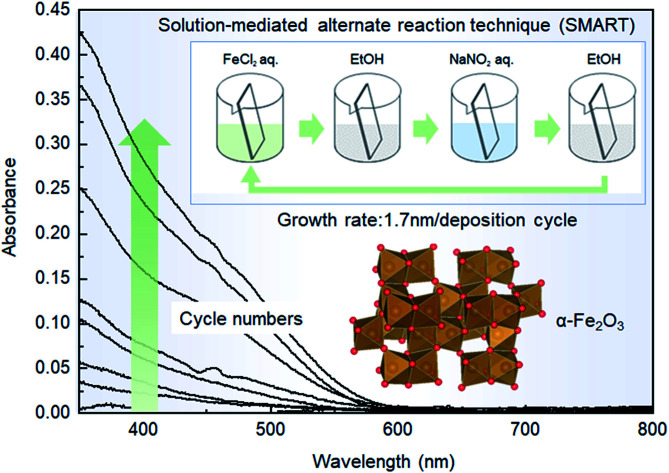

## Introduction

Ceramics coating is a core technology that affords various functions to a substrate material. Metal oxides are particularly attractive for applications in anticorrosion,^1^ catalysis,^2^ sensing,^3^ energy storage^4^ and conversion,^5^ optics,^6^ and electronics.^7^ In an industry context, oxide materials are deposited by vacuum phase deposition techniques such as chemical vapor deposition,^8^ pulsed laser deposition,^9^ and sputtering.^10^ Solution deposition techniques such as sol–gel,^11^ electrodeposition,^12^ and chemical bath-deposition (CBD) methods^13^ are potential alternatives to the above-mentioned vacuum processes as they utilize inexpensive and less toxic solution precursors as well as ambient pressure for oxide deposition, hence making the fabrication more environmentally friendly and more cost-effective.

Moreover, a solution process enables the direct deposition of crystalline oxides on the substrate without post-growth annealing. Low-temperature direct deposition is suitable for film formation on low heat-resistant substrates, expanding the potential scope of functional oxide materials as a component in flexible plastic devices and electrochemical devices with indium-tin-oxide substrates. Recent efforts have allowed the fabrication of various oxide (*e.g.*, ZnO,^14^ TiO_2_,^15^ WO_3_,^16^ and SnO_2_ (ref. 17)) *via* aqueous solution routes without the need for a post-annealing treatment. However, so far, the direct solution routes have been adopted for tiny members in the broad family of metal oxides. The major difficulty lies in the growth mechanism; according to classical aqueous chemistry, the metallic ions (M^*n*+^) in an aqueous solution react with OH^−^ to precipitate hydroxide nucleus on the substrate *via* a heterogenous nucleation process (1):^18^

1 M^*n*+^ + *n*OH^−^ → M(OH)_*n*_

If the stability of hydroxide is sufficiently low for dehydration, the hydroxide spontaneously transforms into an oxide form during deposition (2):^18^2
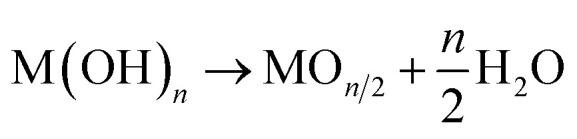


However, hydroxides are often stable so that a post-annealing treatment is inevitable for oxide formation. For example, annealing temperatures over 500 °C are necessary to yield Al_2_O_3_ (ref. 19) and ZrO_2_ (ref. 20) from the hydroxides.

**Fig. 1:**
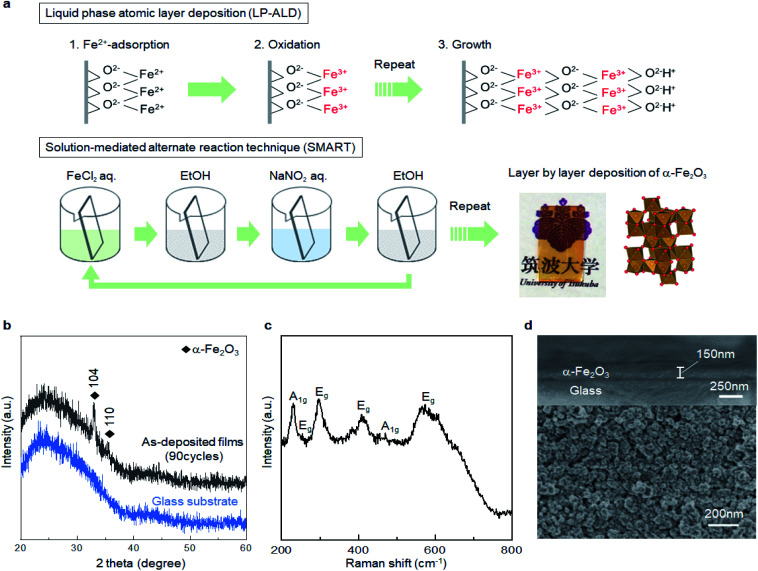
(a) The proposed mechanism of the liquid phase atomic layer deposition (LP-ALD), and the procedure and concept of solution-mediated alternate reaction technique (SMART). (b) XRD pattern, (c) Raman spectrum, and (d) cross-section and surface SEM images of SMART-derived α-Fe_2_O_3_ on a glass substrate deposited after 90 cycles.

This difficulty also applies to α-Fe_2_O_3_, the target material in the present study. Resultant products from classical precipitation reactions between ferric or ferrous precursors with an aqueous base are α-FeOOH, β-FeOOH, γ-FeOOH, σ-FeOOH, and Fe_5_OH_8_·4H_2_O,^21–23^ while one can find no direct solution route to α-Fe_2_O_3_. Nevertheless, Fe_3_O_4_ (magnetite) can be formed *via* a co-precipitation process of ferric and ferrous precursors, even at room temperature.^21^ In other words, Fe–(OH)_2_–Fe bonds can preferentially convert Fe–O–Fe bonding *via* dehydration in an aqueous solution, opening the possibility for direct solution deposition of crystalline α-Fe_2_O_3_ under suitable reaction conditions. In fact, using the above idea, we recently developed a liquid phase atomic layer deposition (LP-ALD) of α-Fe_2_O_3_*via* an onsite oxidation and dehydration pathway by using a spin spray technique.^24^ In this method, a source solution containing Fe^2+^ and an oxidizing solution containing an oxidizer, NaNO_2_, was simultaneously sprayed onto the substrates mounted on a rotating table heated to 95 °C. We propose that the deposition mechanism involved alternative sequences of the absorption of Fe^2+^ ions onto the surface and the subsequent formation of Fe^3+^–oxygen bonds through reactions with the source and oxidizing solutions, respectively (Fig. 1a). We refer to this deposition process as LP-ALD. Such a non-classical LP-ALD strategy may open direct growth pathways to functional metal oxide nanolayers *via* aqueous solution chemistry. However, the spin spray technique is available only in the specialized laboratories.

In the present study, we explore a solution-mediated alternate reaction technique, SMART, to further verify, simplify, and generalize the non-classical LP-ALD reaction pathway (Fig. 1a). Briefly, film deposition in SMART proceeds simply by alternate immersion of the substrate in FeCl_2_ and NaNO_2_ precursor solutions. We demonstrate a primitive beaker process that allows the direct growth of crystalline α-Fe_2_O_3_ films with a growth rate of *ca.* 1.7 nm per cycle. Resultant α-Fe_2_O_3_ thin films exhibit unexpected electrocatalytic activity for oxygen evolution reactions (OER). The origin of catalytic activity comes from the defective nature of SMART-derived α-Fe_2_O_3_ where OH^−^ species are present in the oxide lattice. Sn^4+^-diffusion into the α-Fe_2_O_3_ lattice by annealing and surface modification with Ni(OH)_2_ further enhance the OER activity, which is superior to state of the art α-Fe_2_O_3_-based catalysts.

## Experimental section

### Preparation of the source and oxidizing solutions

FeCl_2_·4H_2_O (Wako Pure Chemical Industries, Ltd., Japan) was used as the metal source precursor. The source solution was prepared by dissolving 20 mM of FeCl_2_·4H_2_O in 50 mL of distilled water. The oxidizing solution was prepared by dissolving 20 mM of sodium nitrite (NaNO_2_, Wako Pure Chemical Industries Ltd., Japan) in 50 mL of distilled water. Nitrogen gas was purged into the solutions at a rate of 1.0 L min^−1^ while stirring in order to prevent the oxidation of the reactants in the solution.

### Deposition process in SMART

A soda-lime glass substrate (25 mm × 25 mm × 1 mm) and a transparent conductive oxide (TCO) coated glass substrate (FTO/ITO, Type-0052, 10 Ω sq^−1^, Geomatec Co., Ltd.) were cleaned in an ultrasonic bath with distilled water, ethanol, and acetone for 10 min each. The glass substrate was further cleaned by treatment in a bath of methanol (Wako Pure Chemical Industries)/HCl (Wako Pure Chemical Industries) (1/1 in volume) for 1 day to obtain a hydrophilic surface, while the TCO substrate was immersed in the 0.1 M HCl (Wako Pure Chemical Industries) for 1 day to obtain a hydrophilic surface.

The surface-modified substrate was first immersed in the source solution heated to 75 °C for 1 min, followed by rinsing with ethanol. Then the substrate was immersed in the oxidizing solution heated at 75 °C for 1 min and rinsed with ethanol again. A series of these operations was repeated from 1 to 90 times to control the film thickness.

### Ni(OH)_2_ surface-modification

α-Fe_2_O_3_ layer deposited on TCO was immersed in 0.1 M Ni(NO_3_)_2_ aqueous solution (Wako Pure Chemical Industries, Ltd., Japan) for 1 min without heating followed by rinsing with water. Then, the substrate was immersed in 1 M KOH solution without heating for 1 min, and then rinsed with water.

### Fabrication of Ni(OH)_2_ layer by a successive ionic layer adsorption and reaction (SILAR)

The surface-modified substrate was first immersed in a 0.1 M Ni(NO_3_)_2_ aqueous solution heated to 75 °C for 1 min, followed by rinsing with water. Then the substrate was immersed in a 1 M KOH aqueous solution heated at 75 °C for 1 min and rinsed with water. The process was repeated 30 times to obtain Ni(OH)_2_ layer.

### Characterization

The crystalline phases of the deposited films were identified by X-ray diffraction (XRD, MultiFlex, Cu Kα, 40 kV and 40 mA, Rigaku). The surface morphologies and textures of the films were observed using a scanning electron microscope (SEM, SU-8020, Hitachi High-Technologies). The elemental distribution was observed an energy dispersive X-ray spectroscopy (EDS, JEOL, JSM-7600). The light absorbance of samples in the ultraviolet-visible (UV-Vis) region was evaluated by the visible absorption spectroscopy (UV-Vis, UV-1280, Shimadzu). X-ray photoelectron spectroscopy (XPS, JPS 9010 TR, JEOL) was conducted to investigate the chemical state of the samples. All measured XPS spectra were calibrated corresponding to the value of the C 1s peak at 284.4 eV using Mg Kα X-ray source with 1253.6 eV. Raman spectroscopy measurements were made LabRam Armis, Horiba Jobin Yvon instrument equipped with 532 nm laser and a microscope to focus the laser light on the film surface.

### Electrochemical and photoelectrochemical measurements

The OER measurements were performed in 1 M KOH aqueous solution using a three-electrode configuration, with a Pt wire counter electrode and an Ag/AgCl, KCl reference electrode. All potentials have been referenced to the reversible hydrogen electrode (RHE) by the expression: *V*_RHE_ = *V*_Ag/AgCl_ + 0.197 V + 0.059 V × pH. The linear sweep voltammograms (LSV) was performed for 20 cycles with a scan rate of 5 mV s^−1^. Photoelectrochemical measurements were performed at the same condition, while visible light (wavelength above 400 nm) was irradiated for the measurements. The 200 W Xeon lamp (Asahi Spectra. Co) was used for the measurements.

## Results and discussion

### Characterization of SMART-derived thin films

The reaction pathway of SMART is designed as follows. In the first step, the substrate is immersed in a FeCl_2_ solution with pH 4 to form a Fe^2+^ adlayer onto the substrate surface. When an oxide surface is negatively charged at the pH, a double layer is formed on the surface where Fe^2+^ forms an inner layer (Stern layer) and the Cl^−^ from the FeCl_2_ precursor forms a charge-balancing outer layer. In the following step, the substrate is rinsed with ethanol so that only the immobile double-layer remains on the substrate surface. Subsequently, the substrate is immersed in the NaNO_2_ solution and heated to 75 °C. The NO_2_^−^ in the solution is diffused onto the surface to oxidize the adsorbed Fe^2+^ to the Fe^3+^ state. At this stage, hydrolysis and dehydration simultaneously occur onsite, resulting in the formation of the first O–Fe–O bonds. In a final step, the substrate is rinsed again to remove the ions from the diffusion layer. In principle, the repletion of these cycles leads to a layer-by-layer deposition of the α-Fe_2_O_3_ layer.

Fig. 1b shows the X-ray diffraction pattern from the sample prepared by the SMART on the glass substrate after 90 cycles of deposition. The pattern displays broad peaks from the glass substrate and sharp peaks corresponding to 104 and 110 reflections of the α-Fe_2_O_3_ phase (JCPDS 33-0664) without an impurity phase such as Fe(OH)_2_, Fe(OH)_3_, FeOOH, Fe_3_O_4_, and γ-Fe_2_O_3_. According to Scherrer's equation using a 104 peak, the crystallite size is calculated to be 47.4 nm.^25^ Raman spectroscopy was employed to further investigate the phase purity of the resultant films. As shown in Fig. 1c, all the detected peaks are assigned as two A_1g_ modes (231 and 473 cm^−1^) and four E_g_ modes (255, 297, 411, and 576 cm^−1^),^26,27^ in support of the film being composed of α-Fe_2_O_3_ domains. Note that Raman spectra up to 1400 cm^−1^ shown in Fig. S1, ESI† also exclude the formation of iron-based impurity phases.^28^

Fig. 1d displays the cross-sectional and surface SEM images of the SMART-derived α-Fe_2_O_3_ film. The cross-sectional image reveals the formation of a dense layer with a relatively uniform thickness of 150 nm on average. Thus, the growth rate can be estimated at approximately 1.7 nm per deposition cycle, if the film thickness linearly increases in each deposition cycle. The surface image presents a continuous film consisting of dense grains with an average size of *ca.* 50 nm. The grain size roughly matches the calculated crystallite size (47.4 nm) from the XRD pattern, indicating that each grain consists of a single crystalline domain. Indeed, the SEM observation with a lower magnification confirms that the film is free from cracks (Fig. S2, ESI†). Owing to the high uniformity, the film was transparent with a yellowish red color, as shown in the inserted photograph in Fig. 1a. The film exhibited good adhesion to the substrate after scotch tape testing, indicating the presence of chemical bonds at the interface between the substrate and the α-Fe_2_O_3_ layer.

### Growth rate and mechanism of SMART process

**Fig. 2:**
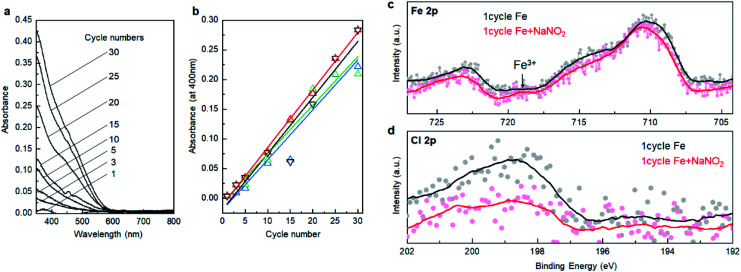
(a) UV-Vis spectra of SMART-derived α-Fe_2_O_3_ obtained after 1, 3, 5, 10, 15, 20, 25, and 30 deposition cycles, (b) plots of absorption intensity at 400 nm *versus* deposition cycles, where the ∇ and Δ symbols denote the plots from the sample deposited on glass and TCO substrates, respectively. (c) Fe 2p and (d) Cl 2p XPS spectra of the deposited layer after the first step (black) and second step (red) in the first deposition cycle.

We verify the growth mechanism by monitoring the change of UV-Vis spectra with an increase in the number of deposition cycles. Fig. 2a shows a change in the UV-Vis absorption spectra after 1, 3, 5, 10, 15, 20, 25, and 30 cycles on the glass substrate. In general, the absorption gradually intensified with the number of deposition cycles, while the spectral feature, *e.g.*, absorption onset, was not largely changed after at least 3 cycles, indicating that the α-Fe_2_O_3_ layer was deposited throughout the cycles. Fig. 2b displays plots of absorption intensity at 400 nm *versus* the number of deposition cycles. The plots were fitted with lines at a slope of *ca.* 0.01 per cycle. Note that the absorption coefficient for 90 cycles of deposited α-Fe_2_O_3_ was 1.12 at 400 nm (Fig. S3, ESI†). From this value, the slope was calculated to 0.012 per cycle, which accords well to the slope value obtained from up to 30 cycles. Thus, the UV-Vis absorption data supports that the α-Fe_2_O_3_ film is deposited in a layer-by-layer manner. The calculated crystallite size, 47.4 nm from the XRD pattern (Fig. 1b), was 28 times larger than the growth rate (*ca.* 1.7 nm). Thus, the Fe^2+^ species in the Stern layer was mainly consumed for crystal growth rather than the heterogeneous nucleation process. We confirm that the slope value that fitted with the UV-Vis data was unchanged when the TCO substrate was used as the substrate (Fig. 2b). This result is reproducible. The thickness of a α-Fe_2_O_3_ layer on an TCO was observed to approximately 50 nm after 30 deposition cycles (Fig. S4, ESI†). This further confirms that the growth rate was about 1.7 nm per cycle.

We employed X-ray photoemission spectroscopy (XPS) to detect the changes in surface states of the first cycle. Fig. 2c displays Fe 2p XPS spectra of the deposited layer after the reaction with FeCl_2_ followed by the rinse step, and one subsequently reacted with the NaNO_2_ solution followed by a second rinse step. Note that the Sn 3p_3/2_ background signal was extracted from the as-obtained data to better understand the Fe 2p spectra see Fig. S5, ESI.† The Fe 2p spectra involve multi-components including Fe 2p_1/2_, Fe 2p_3/2_, and their satellite peaks, while it can be quantitively described that the peaks shifted toward the higher energy side after reaction with NaNO_2_. Indeed, a peak at around 719 eV, attributable to a Fe 2p_1/2_ satellite of Fe^3+^, was pronounced after the reaction.^29,30^ These changes demonstrate that the oxidation of Fe^2+^ occurred by reactions with the NaNO_2_ solution. Besides, the intensity of the Cl 2p peak (Fig. 2d) decreased after the second step. This supports the notion that the Cl^−^ ions involved in the outer layer of the double-layer were replaced by O^2−^ or OH^−^ species binding with Fe^3+^ after the oxidation step. Thus, all the analytical results support that the α-Fe_2_O_3_ layer could be deposited, according to the designed SMART concept (Fig. 1a). The direct formation of α-Fe_2_O_3_ demonstrates that controlling surface redox reactions in the growth process plays a critical role in crystallization. The idea would be applied to bring intriguing aqueous routes to crystalline metal oxides based on multivalent metallic components such as Cu^1+/2+^, Co^2+/3+^, and Mn^2+/3+/4+^.

### Electrocatalytic properties of SMART-derived thin films

**Fig. 3:**
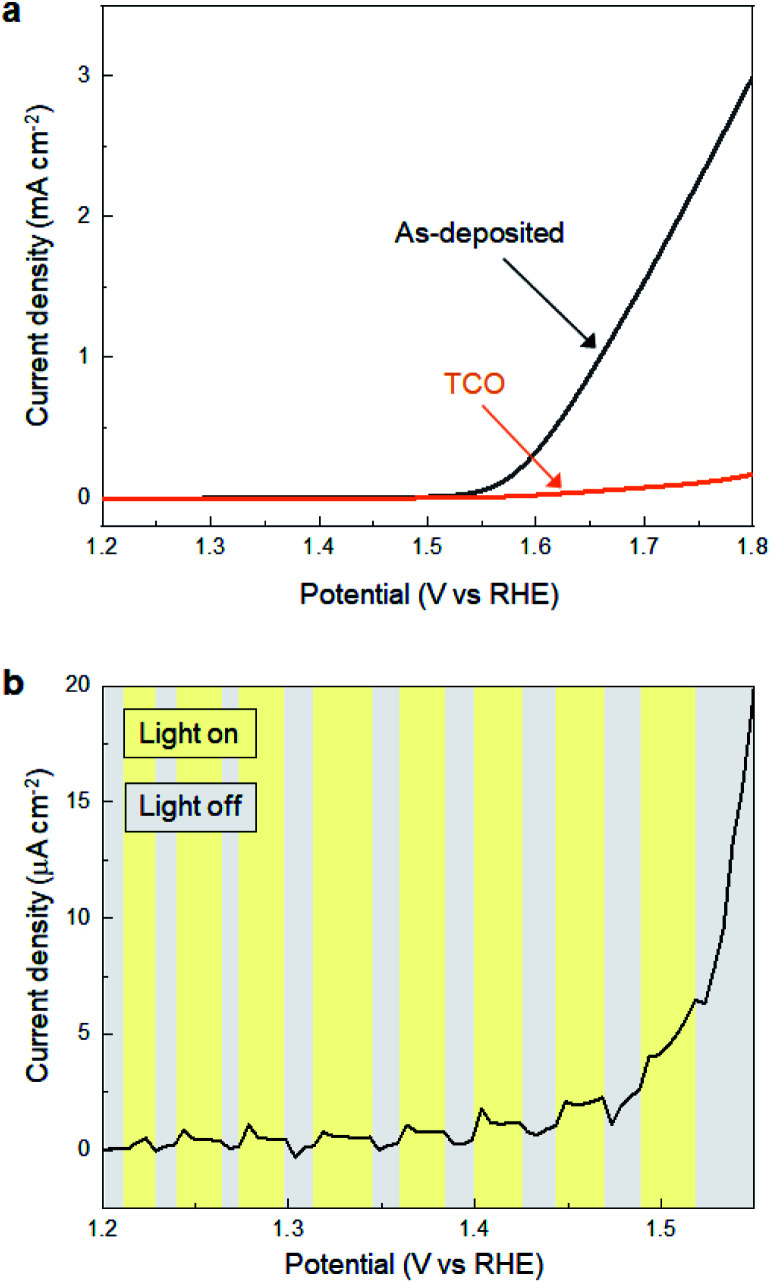
(a) LSV curves for SMART-derived α-Fe_2_O_3_ obtained after 30 deposition cycles on an TCO substrate and bare-TCO. (b) The effects of photoirradiation on the current density for SMART-derived α-Fe_2_O_3_ obtained after 30 deposition cycles on an TCO substrate.

Subsequently, we investigated the electrochemical and photo-electrochemical catalytic performance of SMART-derived α-Fe_2_O_3_ for an oxygen evolution reaction (OER) to find any structure–performance correlations. As is well known, OER is the essence of renewable fuel generation in water electrolysis, and development of stable, cost-effective, and environmentally-friendly OER catalysts is a key challenge.^31–35^ This sheds light on α-Fe_2_O_3_ as one of the most suitable materials.^36,37^Fig. 3a shows plots of linear sweep voltammograms (LSV) of a α-Fe_2_O_3_ deposited TCO substrate after 30 cycles of SMART along with a bare-TCO reference. In this case, the measurements were performed without light-irradiation. A bare TCO shows a tiny cathodic current (0.18 mA cm^−2^ at 1.8 V), while a current of more than one order of magnitude higher is gained at the same potential after α-Fe_2_O_3_ deposition. The overpotential is about 390 mV at 0.5 mA cm^−2^.

We also performed electrochemical measurements under light-irradiation to investigate catalytic activity of photo-electrochemical water oxidation (Fig. 3b). As a result, a tiny increase of cathodic current in the μA cm^−2^ range was observed, which demonstrates that most photogenerated electron/hole pairs were expensed for the recombination pathways rather than for water oxidation. To our knowledge, there has been no report of α-Fe_2_O_3_-based materials simultaneously exhibiting good electrocatalytic activity (measured without light-irradiation) and photo-electrocatalytic activity (measured with light-irradiation) for water oxidation. This is most probably because they are in a trade-off relationship; namely, defects increase the electron conductivity required for the former catalysis, while they induce recombination pathways for the photogenerated carriers that are avoided for the latter. Considering that SMART was conducted at a low temperature of 75 °C, a SMART-derived α-Fe_2_O_3_ layer would be more defective than those synthesized by high-temperature methods, which could be the main reason for the remarkable catalytic activity of SMART-derived α-Fe_2_O_3_.

**Fig. 4:**
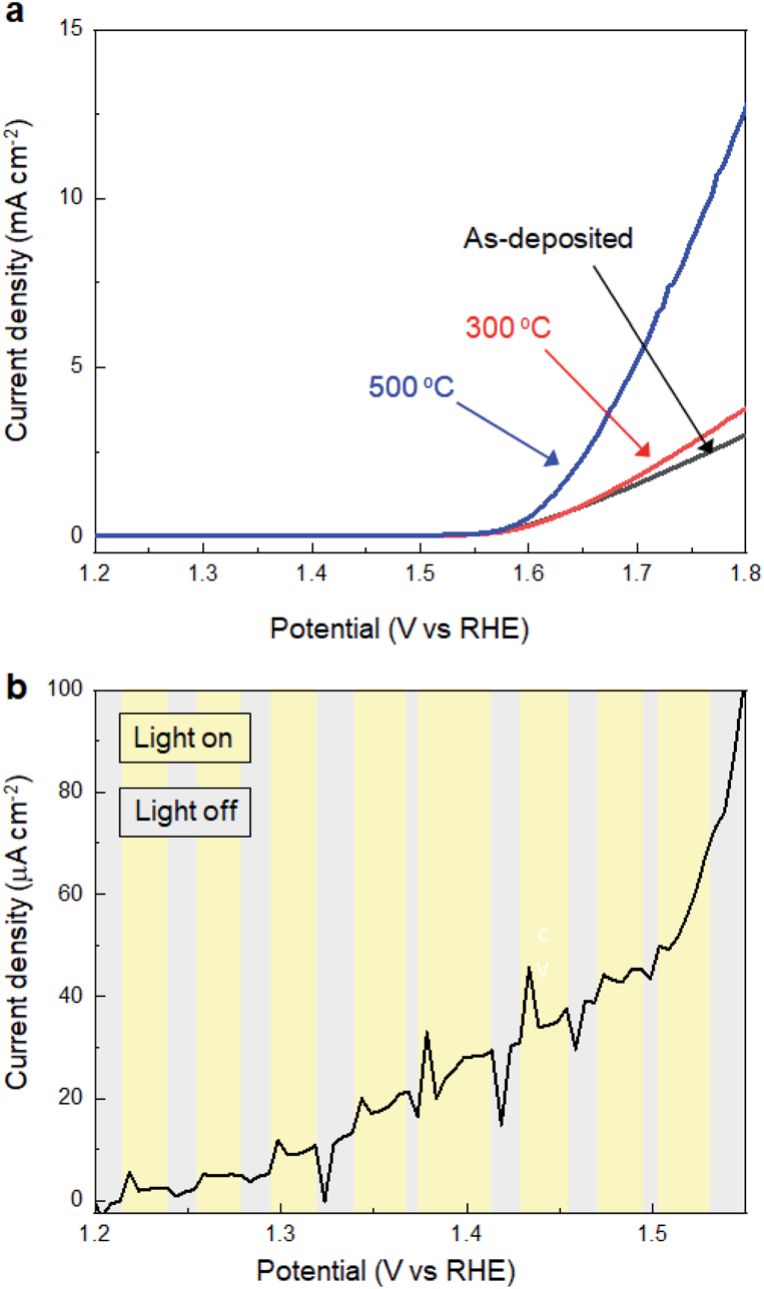
(a) LSV curves for SMART-derived α-Fe_2_O_3_ obtained after 30 deposition cycles on an TCO substrate; as-deposited, and subsequently annealed at 300 °C and 500 °C. (b) The effects of photoirradiation on the current density for SMART-derived α-Fe_2_O_3_ obtained after 30 deposition cycles on an TCO substrate (annealed 500 °C).

Subsequently, we investigated the effects of thermal annealing in air on the electrochemical properties of SMART-derived α-Fe_2_O_3_ to understand the correlation between the catalytic activity and local structures in α-Fe_2_O_3_ layers. Fig. 4a shows the LSV curves of the SMART-derived α-Fe_2_O_3_ layer, as-deposited and subsequently annealed at 300 °C and 500 °C. After annealing at 300 °C, the cathodic current density increased slightly. The catalytic activity was significantly enhanced with the overpotential of 370 mV at 0.5 mA cm^−2^ after annealing at 500 °C. We assumed that the activity would decrease after the annealing in air due to the elimination of defects. However, the trend of the experimental results was the opposite of our expectations, indicating that the α-Fe_2_O_3_ layer remained defective after annealing. In fact, the photo-response remained negligible after annealing at 500 °C (Fig. 4b).

**Fig. 5:**
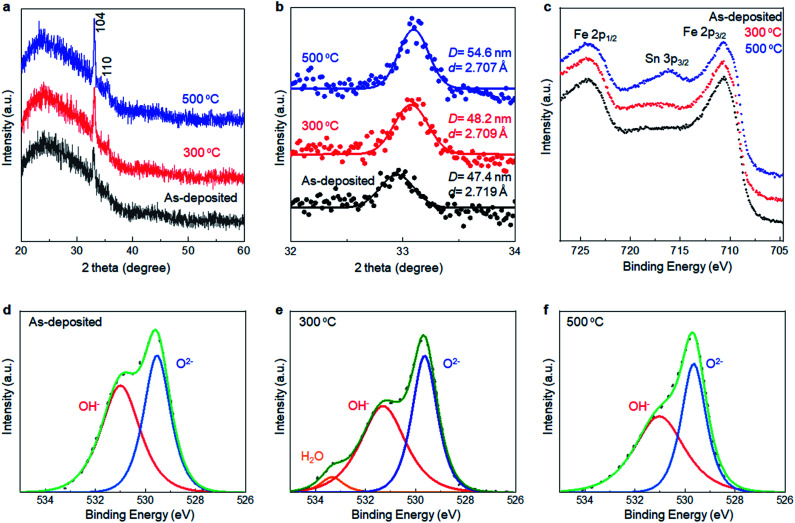
(a) Total and (b) Selected angle XRD patterns of the SMART-derived α-Fe_2_O_3_ deposited on a glass substrate. (c) Fe 2p XPS spectra of the SMART-derived α-Fe_2_O_3_ deposited on an TCO substrate, as-deposited, and subsequently annealed at 300 °C and 500 °C. O 1s XPS spectra of the SMART-derived α-Fe_2_O_3_ deposited on an TCO substrate, (d) as-deposited, and subsequently annealed at (e) 300 °C and (f) 500 °C.

XRD analyses were performed to collect information about the local structure changes of α-Fe_2_O_3_ layers after annealing (Fig. 5a and b). First, no additional peaks emerged after annealing up to 500 °C, supporting that the as-deposited films were mainly composed of crystalline α-Fe_2_O_3_. Second, the average crystallite size slightly increased to 54.6 nm from 47.4 nm after annealing at 500 °C. Third, the *d*-value calculated from the 104 peak position corresponded to 2.719 Å, 2.709 Å, and 2.707 Å for the as-deposited film and ones subsequently annealed at 300 °C and 500 °C, respectively, while the *d*-value obtained from a reference bulk crystalline α-Fe_2_O_3_ was 2.703 Å (Fig. S6, ESI†). Thus, the lattice expansion occurred in as-deposited α-Fe_2_O_3_, and the lattice shrank to the bulk value after the annealing.

XPS was performed to detect the change in chemical states of the α-Fe_2_O_3_ after annealing. Fig. 5c–f show Fe 2p and O 1s spectra of SMART-derived α-Fe_2_O_3_ with and without annealing at 300 °C and 500 °C. In the Fe 2p spectra, a Sn 3p_3/2_ peak located at 716 eV appeared after annealing at 500 °C. It was also found that Sn 3d peaks in the wide-scan spectrum were pronounced after the annealing (Fig. S7, ESI†). Thus, Sn^4+^ ions, involved in the conducting substrate, would be thermally diffused in the α-Fe_2_O_3_ lattice. In fact, this phenomenon can be found in the literatures.^38–40^ Sn^4+^ exhibits a similar ionic radius and Pauling electronegativity to Fe^3+^ ions, which facilitates the substitution of Fe^3+^ to Sn^4+^ in α-Fe_2_O_3_. Importantly, Sn^4+^-doping has been found to be an effective approach for tailoring the electronic properties of α-Fe_2_O_3_.^41–43^ In the present case, the catalytic activity was likely enhanced by an increased electron conductivity by Sn^4+^-doping.^43^ Two binding energies of O 1s (529.5 eV and 531.0 eV) were assigned to the O^2−^ and OH^−^, respectively.^44,45^ The ratio of OH^−^/O^2−^ decreased after annealing at 300 °C. Thus, we suggest that the observed lattice expansion in the as-deposited α-Fe_2_O_3_ layer was caused by the OH species.^46,47^ Finally, the intensity of the Cl 2p was quite weak in the as-deposited sample. The peak was almost undetectable after annealing at 300 °C, excluding the significant contributions of Cl^−^ to change the catalytic activity upon annealing (Fig. S8, ESI†).

### Enhanced catalytic activity at Ni(OH)_2_/α-Fe_2_O_3_ heterointerface

**Fig. 6:**
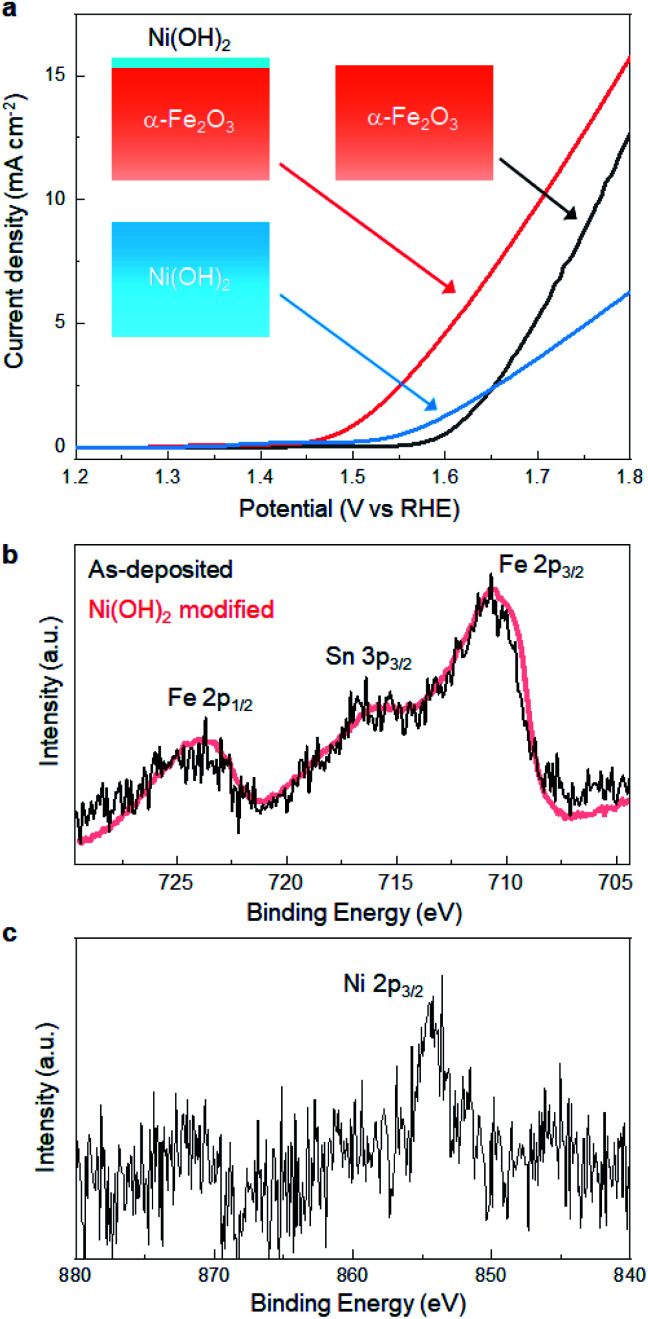
(a) LSV-curves of the SMART-derived α-Fe_2_O_3_ (annealed at 500 °C) before/after Ni(OH)_2_ surface-modification and SILAR-derived Ni(OH)_2_ (b) Fe 2p and (c) Ni 2p XPS spectra of the SMART-derived α-Fe_2_O_3_ (annealed at 500 °C) after Ni(OH)_2_ surface modification.

We revealed that defect-engineering by annealing is effective in enhancing the electrocatalytic activity of α-Fe_2_O_3_. Here, we further extended the LP-ALD concept to tailor the catalytic activity, where the Ni(OH)_2_ layer was decorated onto the surface of the α-Fe_2_O_3_ film after annealing at 500 °C. The Ni(OH)_2_ layer was deposited SILAR method,^18,48^ referred to as the most relevant deposition technique to SMART. In SILAR, metal ions were adsorbed onto the surface followed by rinsing with water. In the next step, metal cations reacted with an alkaline solution to form a metal hydroxide layer *via* classical precipitation reactions (eqn (1)). In fact, this attempt significantly improved the OER activity; as shown in Fig. 6a, the overpotential was lowered to 250 mV at 0.5 mA cm^−2^ after the Ni(OH)_2_-modification. The overpotential at the same current density was *ca.* 50 mV lower than those from the best α-Fe_2_O_3_-based catalysts, Ni- or Zn-doped α-Fe_2_O_3_, reported so far.^36^ No degradation of catalytic performance was observed after 100 scans, which indicated the catalytic durability. The Ni(OH)_2_-modified α-Fe_2_O_3_ showed better catalytic activity than SILAR-derived Ni(OH)_2_, where the overpotential of a Ni(OH)_2_ layer obtained after 30 deposition cycles was 320 mV at 0.5 mA cm^−2^. Fig. 6b and c show the Fe 2p and Ni 2p XPS spectra of Ni(OH)_2_-modified α-Fe_2_O_3_, respectively. There is no remarkable change in the features of the Fe 2p spectra, indicating that the Fe–O–Fe framework containing oxygen vacancies was not altered by Ni(OH)_2_-modification. Based on the relative peak intensity of the Ni 2p and Fe 2p spectra, the Ni : Fe atomic ratio is approximately 1 : 3. Considering the analytical depth of the XPS (*ca.* 4 nm), the thickness of the Ni(OH)_2_ layer is estimated to be 1 nm. Besides, SEM-EDX analysis revealed that Ni signal was homogenously detected on the whole surface of the α-Fe_2_O_3_ layer, while there was no morphological change on the surface (Fig. S9, ESI†). In addition, no additional reflection peaks from Ni-based phases were detected in the XRD pattern of the Ni(OH)_2_-modified sample (Fig. S10, ESI†). This indicates that particulate Ni(OH)_2_ was not formed, while Ni(OH)_2_ would be uniformly formed on the α-Fe_2_O_3_ surface. These results support that catalytic activity was modified through the formation of Ni(OH)_2_/α-Fe_2_O_3_ heterointerface.

Finally, for perspective, we propose that the LP-ALD process could provide a platform to create artificial two-dimensional (2D) heterostructures. 2D heterostructures such as BaTiO_3_/SrTiO_3_ superlattice were initially fabricated by a vacuum process,^49^ and recently, hetero-assembly of 2D nanomaterials such as graphene, as well as 2D transition metal dichalcogenides and 2D oxides have attracted considerable attention for tuning functionalities by interface coupling.^50,51^ Although LP-ALD, including SILARs and solution-ALD,^52^ has only been employed for the deposition of inorganic layers with single components, the layer-by-layer deposition principle is applicable to produce such 2D heterostructures. The Ni(OH)_2_/α-Fe_2_O_3_ heterointerface with excellent OER activity, found in the present study, partially demonstrate the above strategy. However, a true understanding of the enhancement of the heterointerface remains challenging because of the complex nature of the surface system, and thus is beyond the scope of this current study. For example, we found that enhancement of OER activity was less significant, when Ni(OH)_2_ was deposited on the as-deposited α-Fe_2_O_3_ layer (Fig. S11, ESI†). Presently, we expect that the periodical 2D heterostructures, such as alternately stacked Ni(OH)_2_/α-Fe_2_O_3_ layers, would serve rich chemistry, affording superior catalytic activity. This will be the target of our next study.

## Conclusion

In conclusion, we established SMART for the direct solution deposition of α-Fe_2_O_3_ layers on oxide substrates. This method yielded a α-Fe_2_O_3_ layer with a 150 nm thickness and a crystalline size of 47.4 nm after 90 deposition cycles. The growth rate was *ca.* 1.7 nm per deposition cycle, in which Fe^2+^ cations in a Stern layer were oxidized by NaNO_2_ to form Fe^3+^ followed by consumption by crystal growth. Thus, the designed reaction route for the α-Fe_2_O_3_ layer was experientially demonstrated. OH^−^ ligands were introduced in the lattice of α-Fe_2_O_3_ crystallites, probably because of the low-temperature aqueous process. The defective feature of SMART-derived α-Fe_2_O_3_ activated and deactivated electrochemical and photoelectrochemical activity for water oxidation, respectively. The annealing in air introduced the Sn^4+^ ions in the α-Fe_2_O_3_ layer by the thermal diffusion from the substrate, which enhanced the electrocatalytic activity. Finally, we found that Ni(OH)_2_/α-Fe_2_O_3_ heterointerface provided excellent OER activity, which would be crucial to develop stable, cost-effective, and environmentally-friendly OER catalysts.

## Conflicts of interest

There are no conflicts to declare.

## Supplementary Material

NA-002-D0NA00345J-s001
