# Supplementary material for: Solution-mediated nanometric growth of α-Fe2O3 with electrocatalytic activity for water oxidation
Source: Nanoscale Adv. 2020 Jul 20;2(9):3933–41. doi: 10.1039/d0na00345j (PMC9417511; doi:10.1039/d0na00345j)
Supplement: NA-002-D0NA00345J-s001 [file NA-002-D0NA00345J-s001.pdf]

Electronic Supplementary Material (ESI) for Nanoscale Advances.
This journal is © The Royal Society of Chemistry 2020

Supporting Information

Solution-mediated Nanometric Growth of α-Fe_2O_3 with Electrocatalytic Activity for Water Oxidation

Asako Taniguchi[a,b*], Yuta Kubota[c], Nobuhiro Matsushita[c], Kento Ishii[b], Tetsuo Uchikoshi[b]

[a]Graduate School of Pure and Applied Sciences, University of Tsukuba, 1-1-1 Tennodai, Tsukuba, Ibaraki 305-8573, Japan

[b]Research Center for Functional Materials, National Institute for Materials Science (NIMS), 1-2-1 Sengen, Tsukuba, Ibaraki 305-0047, Japan

[c]Department of Materials Science and Engineering, School of Materials and Chemical Technology, Tokyo Institute of Technology, 2-12-1 Ookayama, Meguro-ku, Tokyo 152-8550, Japan

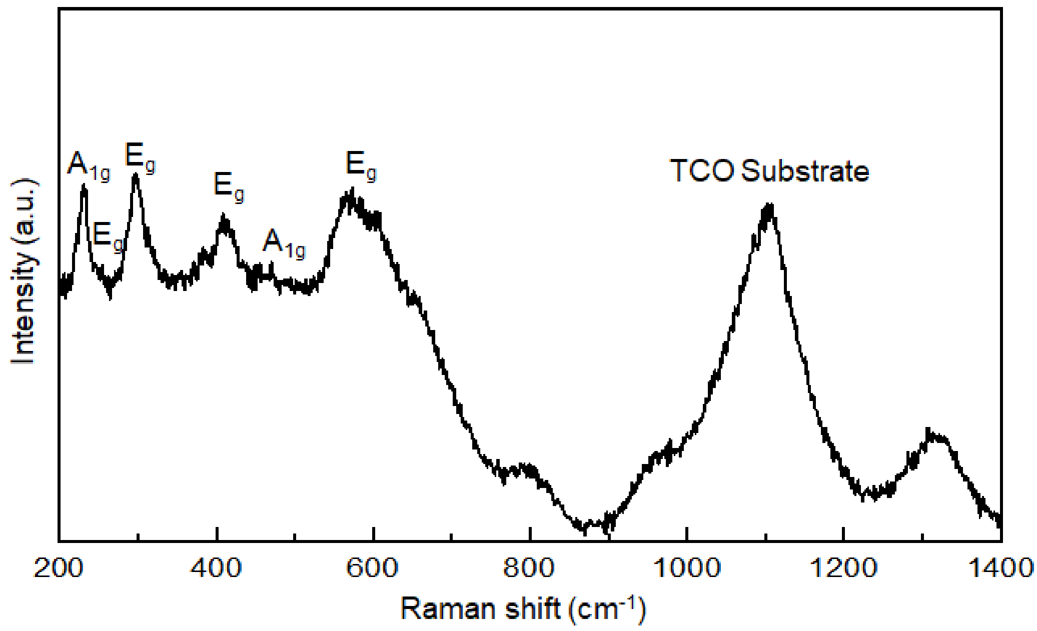

Figure S1. Wide-range Raman spectrum of SMART-derived α-Fe_2O_3 on a glass substrate deposited after 90 cycles.

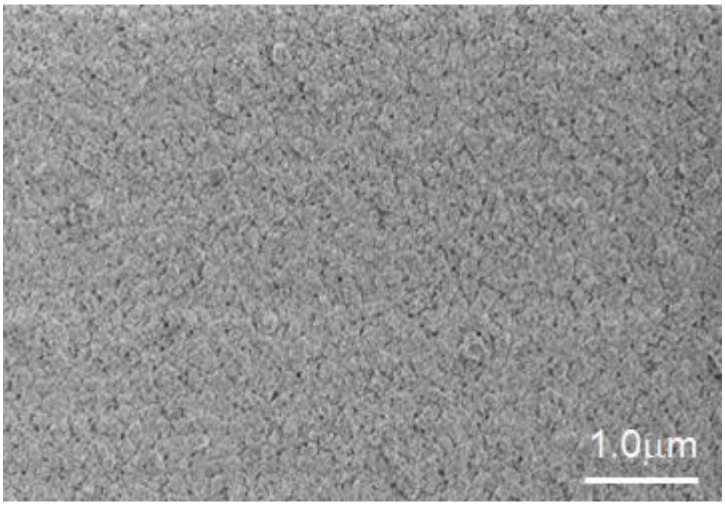

Figure S2. Low magnification SEM image of SMART-derived α-Fe_2O_3 layer on a glass substrate after 90 deposition cycles.

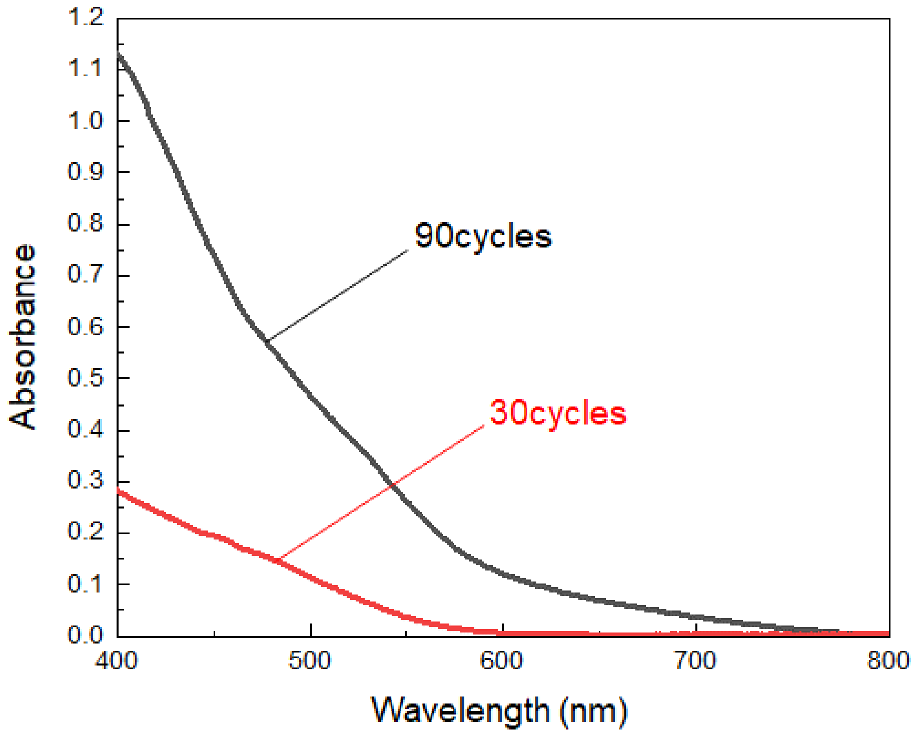

Figure S3. UV-vis spectra of SMART-derived α-Fe_2O_3 layer on a glass substrate after 30 and 90 deposition cycles.

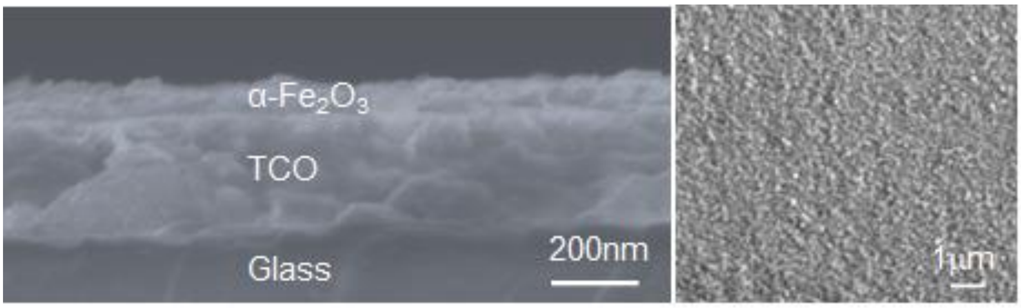

Figure S4. SEM images of SMART-derived α-Fe_2O_3 on an TCO substrate deposited after 30 cycles.

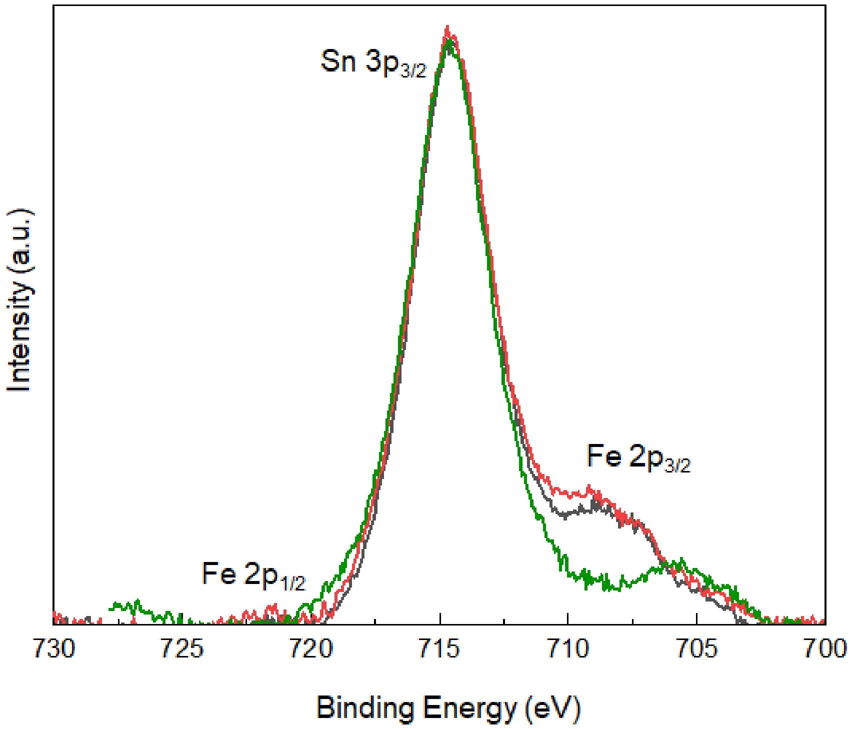

Figure S5. As-measured Fe 2p XPS spectra after the first step (black) and second step (red) in the first deposition cycle, and Sn 3p spectra of a bare TCO substrate (green).

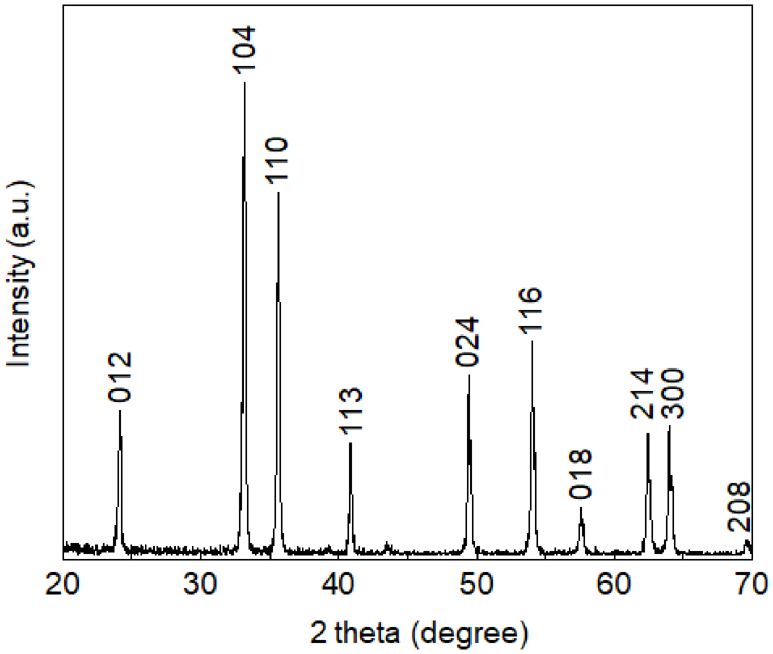

Figure S6. XRD pattern of reference α-Fe_2O_3 powder.

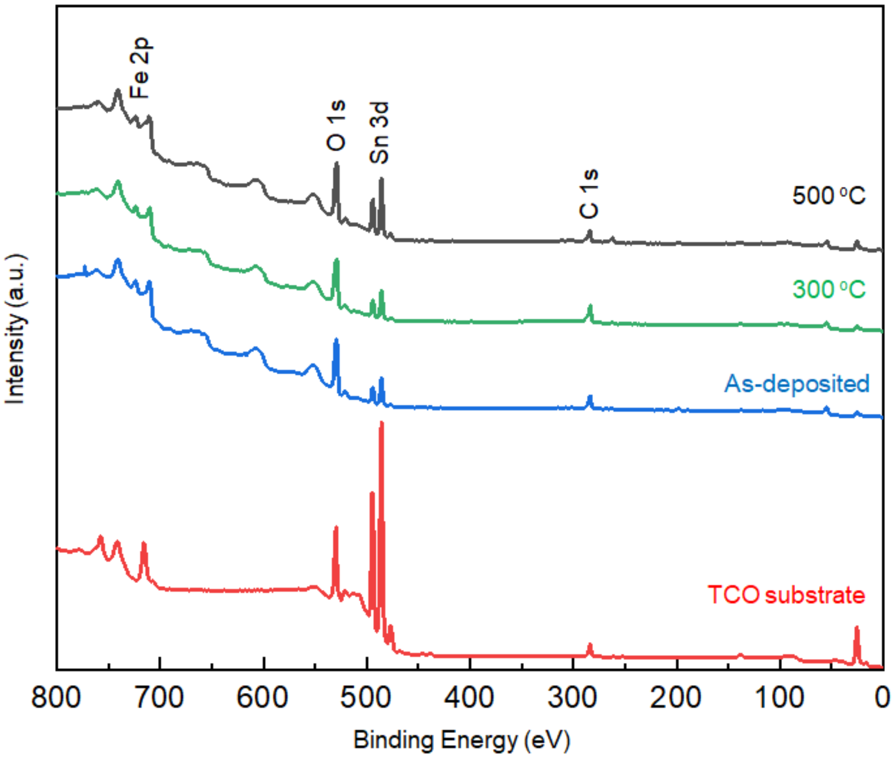

Figure S7. Wide-scan XPS spectrum of the SMART-derived α-Fe_2O_3 deposited on an TCO substrate, as-deposited, and subsequently annealed at 300 °C and 500 °C. Wide-scan XPS spectrum of TCO substrate is shown as a reference.

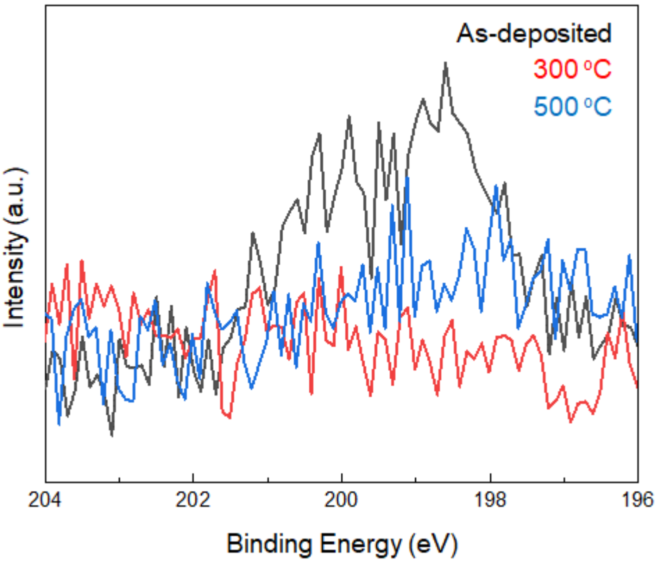

Figure S8. Cl 2p spectra of the SMART-derived α-Fe_2O_3 deposited on an TCO substrate, as-deposited, and after annealing at 300 °C and 500 °C.

(a)

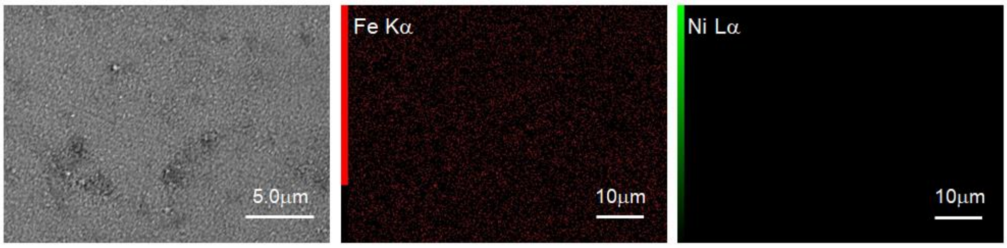

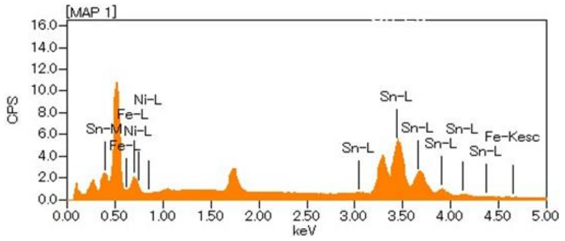

Element	Wt %	At %	σ	Net Int.	K-Ratio	Line
Fe	15.90	28.66	0.18	28265	0.0202418	K
Ni	0.02	0.03	0.08	112	0.0000078	L
Sn	84.08	71.31	0.24	653167	0.0804101	L
Total	100.00	100.00				

(b)

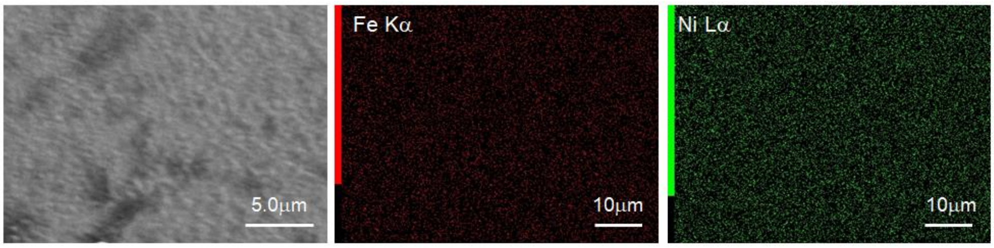

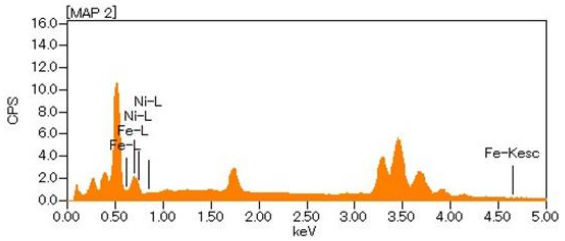

Element	Wt %	At %	σ	Net Int.	K-Ratio	Line
Fe	14.91	26.91	0.18	28951	0.0188464	K
Ni	0.98	1.69	0.08	6614	0.0004165	L
Sn	84.10	71.40	0.23	712416	0.0797225	L
Total	100.00	100.00				

Figure S9. SEM images and Fe/Ni distributions obtained by EDS elemental mapping of SMART-derived α-Fe_2O_3 (a) before and (b) after $Ni(OH)_2$ surface-modification.

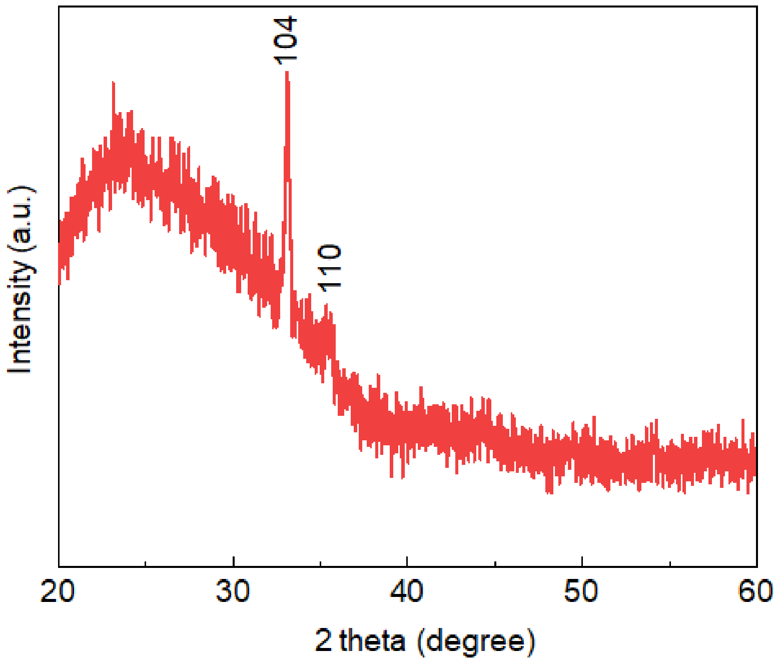

Figure S10. XRD pattern of the SMART-derived α-Fe_2O_3 (annealed at 500 °C) after $Ni(OH)_2$ surface-modification. Note that α-Fe_2O_3 layer was deposited after 90 cycles.

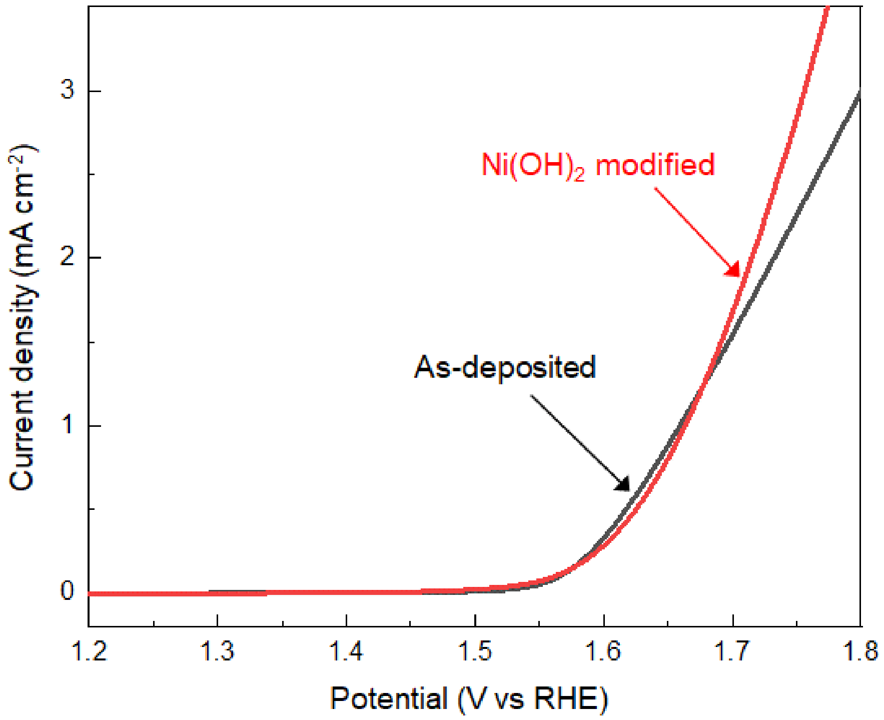

Figure S11. LSV-curves of the SMART-derived α-Fe_2O_3 before/after $Ni(OH)_2$ surface-modification.
